# Effect of Fluoride Doping in Laponite Nanoplatelets on Osteogenic Differentiation of Human Dental Follicle Stem Cells (hDFSCs)

**DOI:** 10.1038/s41598-018-37327-7

**Published:** 2019-01-29

**Authors:** Induvahi Veernala, Jyotsnendu Giri, Arpan Pradhan, Poulomi Polley, Ruby Singh, Sunil Kumar Yadava

**Affiliations:** 1 Department of Biomedical engineering, Indian Institute of Technology Hyderabad, Kandi, Telangana India; 2 Department of Biosciences and Bioengineering, Indian Institute of Technology Bombay, Powai, Mumbai, Maharashtra India

## Abstract

Bioactive nanosilicates are emerging prominent next generation biomaterials due to their intrinsic functional properties such as advanced biochemical and biophysical cues. Recent studies show interesting dose-dependent effect of fluoride ions on the stem cells. Despite of interesting properties of fluoride ions as well as nanosilicate, there is no reported literature on the effect of fluoride-doped nanosilicates on stem cells. We have systematically evaluated the interaction of fluoride nanosilicate platelets (NS + F) with human dental follicle stem cells (hDFSCs) to probe the cytotoxicity, cellular transport (internalization) and osteogenic differentiation capabilities in comparison with already reported nanosilicate platelets without fluoride (NS − F). To understand the osteoinductive and osteoconductive properties of the nanosilicate system, nanosilicate treated hDFSCs are cultured in three different medium namely normal growth medium, osteoconductive medium, and osteoinductive medium up to 21 d. NS + F treated stem cells show higher ALP activity, osteopontin levels and significant alizarin red staining compared to NS − F treated cells. This study highlights that the particles having fluoride additives (NS + F) aid in enhancing the osteogenic differentiation capabilities of hDFSCs thus potential nanobiomaterial for periodontal bone tissue regeneration.

## Introduction

Natural regeneration of bone tissue in our body is limited to small bone defects and relatively large bone defect and their regeneration is highly challenging^1^. Periodontal disease such as periodontitis, progressive loss of alveolar bone and destruction of periodontal ligament, and cementum, culminates in tooth loss in adults and children. Although several treatment modalities have been employed in periodontitis, but regeneration of the total damaged periodontal tissue yet remains as a major unresolved challenge because of the complex periodontium structure^2^.

There is an unmet clinical need of neo bioactive materials for bone tissue regeneration as these materials help in the key process steps for bone regeneration such as manipulate and control the stem cell behavior, osteogenic differentiation and osteoblasts growth. Various types of bioactive materials with clinical relevance have been reported for bone tissue engineering such as bioactive glass (Na_2_O–CaO–SiO_2_ –P_2_O_5_), hydroxyapatite (HA) [Ca_10_(PO_4_)_6_(OH)_2_], β -tricalcium phosphate (TCP) [Ca_3_(PO_4_)_2_], β-wollastonite (CaO-SiO_2_), and Apatite-Wollastonite glass ceramic. However, these materials show limited success for bone tissue engineering, mainly due to lack of osteoinductive properties, poor processing abilities, and insufficient degradation^3,4^.

Bioactive nanosilicates are emerging prominent next generation biomaterials due to their intrinsic functional properties such as advanced biochemical and biophysical cues; owing to their composition, enhanced surface area and adsorption properties. These silicates has already been used in a variety of areas in enhancing matrix properties such as anti fouling surfaces, barrier films^5^, hydrophobic elastomers, hydrogels and in drug delivery applications^6–8^. Very recently, Laponite-XLG nanosilicate nanoplatelets (with empirical formula **Na**^**+**0.7^ **[(Si**_8_ **Mg**_5.5_ **Li**_0.3_**) O**_20_ **(OH)**_4_**]**^−0.7^, BYK Additive and Instrument, USA; abbreviated in the text as ‘Nanosilicate-without-Fluoride (NS − F)) has been reported as a novel material having osteoinductive properties on the stem cells (hBMSC; human bone marrow stem cells, MSC; mesenchymal stem cells) and their osteogenic differentiation^8^. The mechanism of action of these nanosilicate nanoplatelets may be inferred by the cellular and molecular interaction of their corresponding dissolution or dissociation products^8^. Moreover, these nanosilicates gets internalized into the cells through “clathrin–mediated pathway”^9–11^ and are cytocompatible.

Recent studies show interesting dose-dependent effect of fluoride ions on the stem cells where low concentrations of fluoride can positively affect the differentiation of normal human dental pulp stem cells (hDPSCs) *in vitro*, while high fluoride concentration has inhibitory effects on the same cells^12^. Moreover, fluoride ions in low concentrations (0.1–2.0 mgL^−1^) are found to enhance apatite formation by promoting hydrolysis of intermediate octa calcium phosphate (OCP) phase to apatite^13^. Fluoride ions exhibit anticariogenic effect in teeth, aids in remineralisation by interfering in the pellicle and plaque formation and the inhibition of microbial growth and metabolism^13^. Despite of interesting properties of fluoride ions as well as nanosilicate, none of the nanosilicates with fluoride doping has been considered for the studies to understand their effect on stem cells^8,13^. Moreover, the effect of nanoformulation of fluoride on the stem cells is yet to be studied. Our hypothesis is that the nanosilicates with fluoride ion along with other intrinsic silicate ions may be a novel potent bioactive materials for the superior modulation of stem cells proliferation and differentiation. In this study we have used fluoride-dope-nanosilicate platelets, Laponite-XL21 (with empirical formula **Na**^**+**0.7^ **[(Si**_8_ **Mg**_5.5_ **Li**_0.3_**) O**_20_ **(OH)**_4-x_ **F**_x_**]**^−0.7^, from BYK Additive and Instrument, USA; abbreviated in the text as ‘Nanosilicate-with-Fluoride (NS + F)) as a new material to probe the interaction with stem cells and their effect on the modulation of the osteogenic differentiation. To our knowledge, this is the first time we are reporting a fluoride nanosilicate system which aids in osteoinduction of hDFSCs, resulting in the osteogenic differentiation. Moreover, though there is a recent study on the effect of Laponite XLG (NS − F; Nanosilicate platelets without fluoride ions) on hBMSC proliferation and differentiation, the effect on the hDFSCs is yet to be examined.

In this paper we have systematically evaluated the cytotoxicity and osteogenic differentiation capabilities of fluoride nanosilicate platelets (NS + F) in comparison with already reported nanosilicate platelets without fluoride (NS − F) towards human dental follicle stem cells (hDFSCs) in presence of different culture condition (normal: NCM, Osteoconductive medium: OCM and Osteoinductive medium: OIM) to understand the Osteocon nductive and Osteoinductive properties of the nanosilicate system and their interaction with hDFSCs.

## Results

### Structure and physicochemical properties of nanosilicates

**Figure 1:**
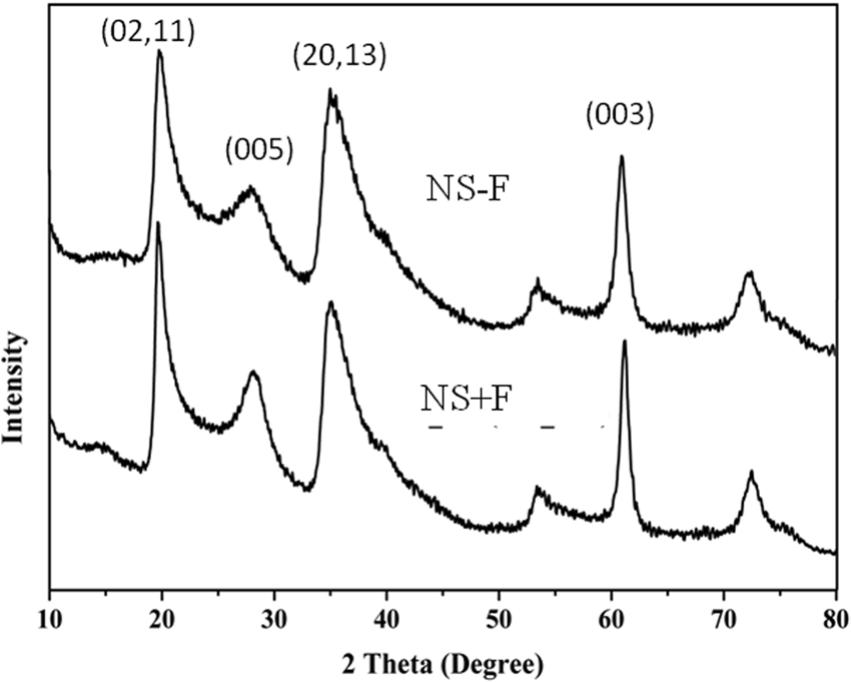
X-ray diffraction pattern of two nanosilicate platelets, NS − F and NS + F.

**Figure 2:**
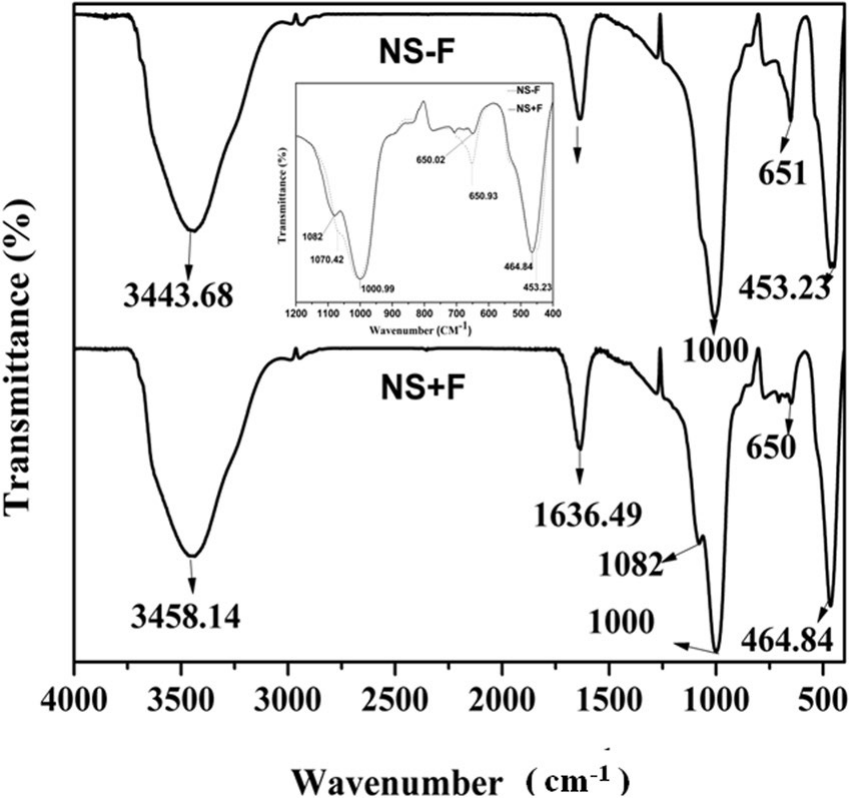
FT-IR spectra of two nanosilicate platelets, NS − F and NS + F. Insert showing enlarged spectra between 400 to 1200 cm^−1^ of both NS.

We have used two laponite nanosilicates (NS) namely NS + F and NS − F, with and without fluoride in their composition respectively for this study. Laponite nanosilicates are a synthetic clay mineral of the smectite group of phyllosilicates composed of layered units of two tetrahedral silica sheets sandwich one Mg^2+^ containing octahedral sheet and are disk-shaped, approximately 25 nm diameter and 0.92 nm height (platelet shape)^8,14^. Based on the information received from BYK, USA, laponite nanosilicates have an empirical formula of Na^+0.7^ [(Si_8_ Mg_5.5_ Li_0.3_) O_20_ (OH)_4-x_ F_x_]^−0.7^ (where x = 0 for NS − F, x ≠ 0 for NS + F and x value not revealed by BYK, USA) where magnesium cations are randomly substituted by lithium ions. Similarly, “OH” groups in Mg^2+^ containing octahedral sheet are randomly replaced by fluoride ions in the fluoride doped laponite nanoslicate (NS + F). However, we characterized these two NS using XRD as well as FT-IR to confirm their structure. Figure 1 shows the XRD pattern of two powder NS particles (NS − F and NS + F). XRD pattern of both NS nanoplates consisted of five clear broad diffraction peaks related to (001), (02,11), (005), (20,13) and (060) plane diffractions. The presence of broad peaks confirms their nanocrystalline nature. There is no difference in the XRD diffraction peaks pattern between the two nanosilicate particles, NS − F and NS + F. Figure 2 shows FT-IR transmission spectra of two NS particles. Two NS show similar FT-IR spectra corresponding to reported laponite nanoparticles (Liponite XLG) spectra^15^ Shoulder peaks between 3500 to 3700 cm^−1^ is related to stretching vibrations of Mg_3_OH, SiOH and Mg_2_LiOH groups, which is overlapped by broad band OH stretching vibrations of H_2_O molecule adsorbed on both NS and KBr (near 3450 cm^−1^). Similarly, band around 1600 cm^−1^ is related to bending mode vibration of H_2_O molecules. The high intensity peak around 1000 cm^−1^ is associated to Si–O stretching vibrations of the tetrahedral sheets. Shoulder peak at 1070 cm^−1^ for NS − F and 1080 cm^−1^ for NS + F are related to Si–O (non-bridging oxygen) vibration. Shifting of peak to higher energy in case of NS + F sample may be due to possible interaction of F in the structure. Peak at 650 cm^−1^ is related to the OH bending vibrations (Mg3OH) (insert spectra)^15^. Moreover, NS − F sample shows higher intensity of this peak compared to NS + F sample. The relatively low intensity of peak at 650 cm^−1^ may be attributed to the presence of lower amount of OH group (attached with Mg^2+^ in the tetrahedral sheet) and confirming the substitution of OH group by F^−^ ion in NS + F samples. The broad peak at 450 cm^−1^ is related to the overlapping bending vibration of Si–O–Mg and Si–O–Si. However, the shifting of these spectra toward higher energy for NS + F samples attribute possible interaction of F^−^ ion in the bending of these bonds.

**Figure 3:**
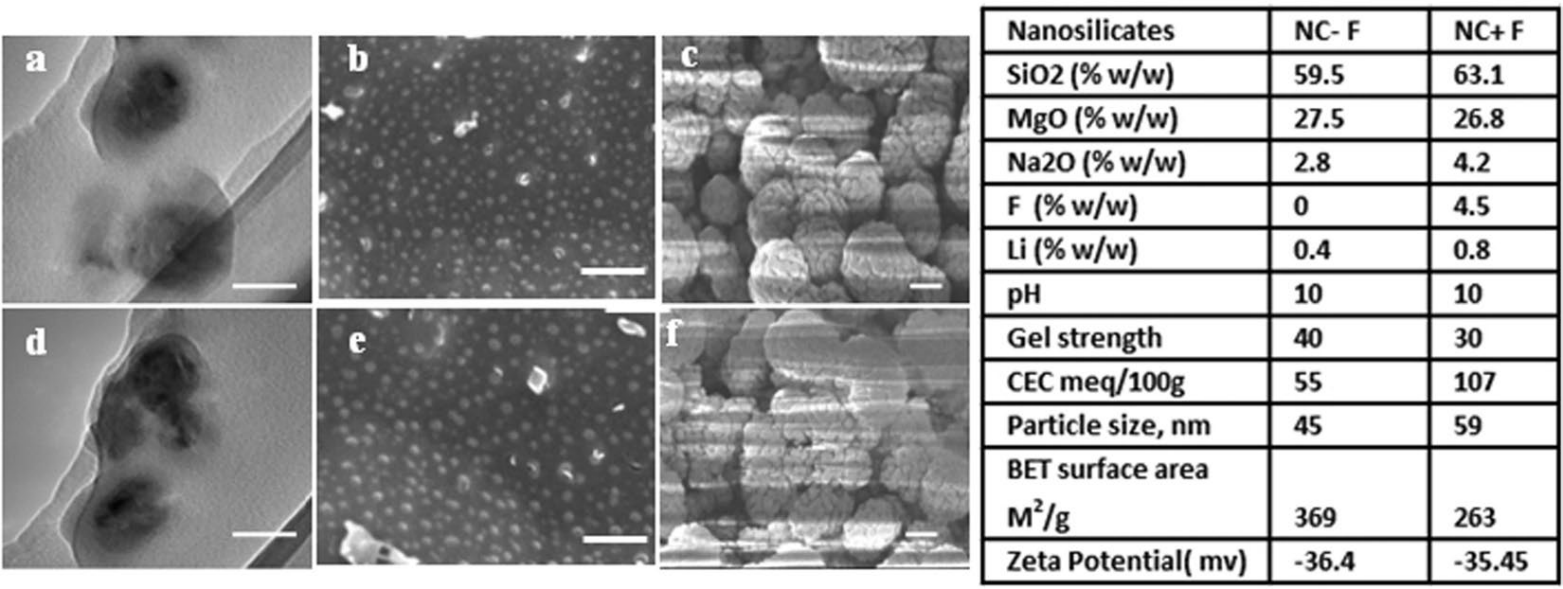
Physicochemical property of nanosilicate platelets (NS). TEM images showing size and morphology of NS − F (**a**) and NS + F (**d**). Cryo-SEM images of NS − F at 100 µg.mL^−1^ (**b**) and 1 mg.mL^−1^ concentration (**c**). Cryo-SEM images of NS + F at 100 µg.mL^−1^ (**e**) and 1 mg.mL^−1^ concentration (**f**). Insert table showing the percent composition, particle size, surface area and zeta potential of NS − F and NS + F nanosilicates. Scale bar is 50 nm for (**a**,**d**), 200 nm for (**b**,**e**) and 300 nm for (**c**,**f**).

Table insert in the Fig. 3 depicts the important physicochemical properties such as composition, particle size, specific surface area, zeta potential of both the NS. Both nanosilicates as expected from their structure have similar negative zeta potential (~36 mV) in DI water (1 mg.mL^−1^) confirming negatively charged surface (face of the nanoplatelet crystal) in the colloidal suspension^16–18^. The high value of the zeta potential provides stable colloidal suspension of NS in their low concentration solution. Figure 3 shows the transmission electron micrograph (TEM) and cryo-scanning electron micrograph (cryo-SEM) of the NS. Typical particles size of NS − F and NS + F are 45 nm and 59 nm respectively (TEM image, Fig. 3a,d). Low concentration (below 1 mg.mL^−1^) of the NS platelets, results in relatively monodispersed particles as visualized in the cryo-SEM Fig. 3(b,e). However, particles agglomerate at higher concentration of NS (1 mg.mL^−1^) as shown in the cryo-SEM Fig. 1(c,f). As low concentration of NS in water as well as cell culture media results in relatively monodispersed nanoplatelets, we have chosen an optimum concentration in between 10 to100 µg.mL^−1^ for the *in vitro* cellular interaction and osteogenic differentiation of NS with hDFSCs.

### Comparative cytotoxicity of nanosilicates

**Figure 4:**
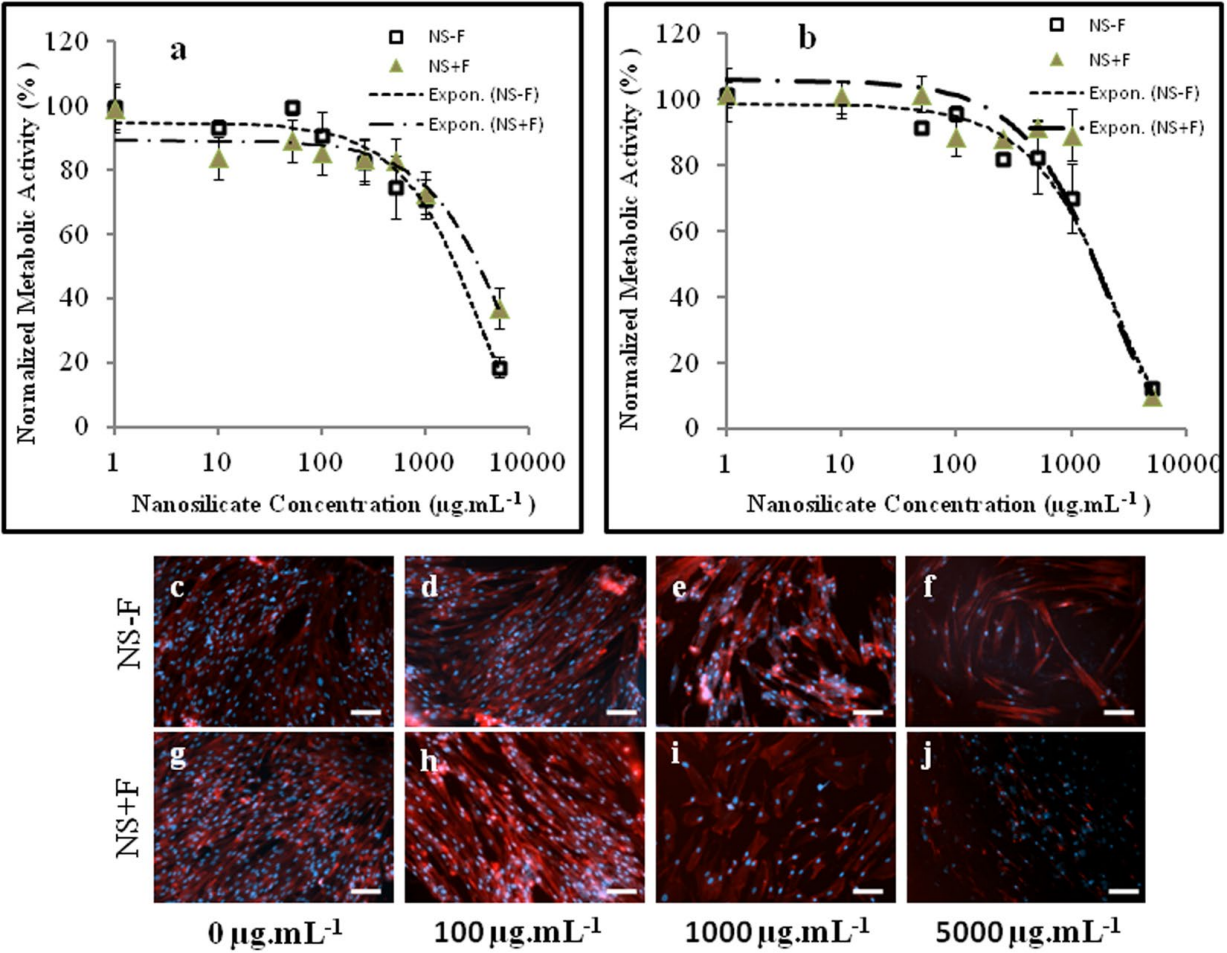
Showing metabolic activity and morphology of hDFSCs after incubation with various concentrations of NS. The percent viability or metabolic activities of hDFSCs in the presence of different concentrations of NS cultured for 24 h (**a**), and 72 h (**b**). Changes in cellular morphology (actin filament arrangement) of hDFSCs when incubated for 48 h in presence of various concentration of NS namely, 0 µg.mL^−1^ (**c**,**g**), 100 µg.mL^−1^ (**d**,**h**), 1000 µg.mL^−1^ (**e**,**i**) and 5000 µg.mL^−1^ (**f**,**j**). Actin filament (red) stain by Alexa flour 546, and nuclei (blue) stained by DAPI. Scale bar is 100 µm.

It is important to study the cytotoxicity of the two nanosilicate platelets comparatively towards stem cells (hDFSCs) for any specific application. We performed MTS assay, and cellular morphology analysis in the presence of different concentration of NS particles (0 µg.mL^−1^ to 5000 µg.mL^−1^) to probe the cytotoxicity of NS. Figure 4(a,b) shows the metabolic activity of hDFSCs cultured up to 72 h in presence of different NS concentration. hDFSCs shows concentration of nanosilicates dependent metabolic activity. 100 percent cell viability is observed till 100 µg.mL^−1^ concentration of both nanosilicates (NS − F and NS + F). The decline in metabolic activity is prominent above the concentration 5000 µg.mL^−1^ of both nanosilicates. The IC_50_ values after 24 h of incubation are 2.2 mg.mL^−1^ and 4.2 mg.mL^−1^ for NS − F and NS + F respectively, whereas after 72 h of incubation these values decrease to 2.0 mg.mL^−1^ and 2.2 mg.mL^−1^ for NS − F and NS + F respectively. The changes in the morphology of hDFSCs cultured in the presence of different concentration of nanosilicate (0 to 5000 µg.mL^−1^) for 48 h, are depicted in Fig. 4(c–j) using fluorescence microscopy. Cells density and morphology of hDFSCs cultured in presence of NS particles (NS − F and NS + F) up to 100 µg.mL^−1^ concentration [Fig. 4(d,h)] are similar with control cells (without NS particles) [Fig. 4(c,g)], confirming the biocompatibility concentration of these NS. At both nanosilicates concentration of 1000 µg.mL^−1^ hDFSCs retain long and spindle fibroblastic morphology similar to the control (without nanosilicate) but decrease in the cell densities in case of NS samples compared to control, confirming the toxicity at 1000 µg.mL^−1^ concentration of NS. The prominent rounded morphology [Fig. 4(f,j)] and relatively low cell density at 5000 µg.mL^−1^ concentration of NS (NS − F and NS + F) confirm the inhibition of cellular functionality and toxicity of NS.

### Live/dead assay

**Figure 5:**
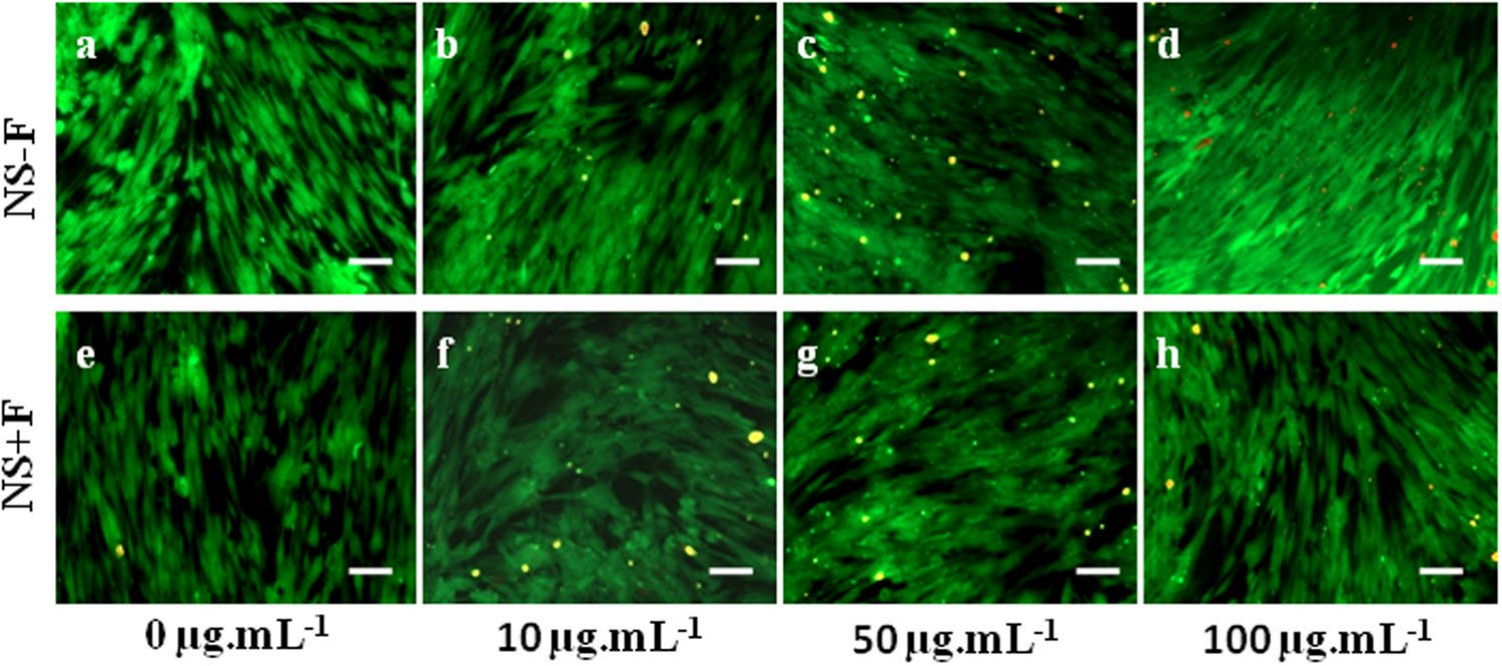
Showing fluorescence microscopy images (FDI/PI) of hDFSCs after incubation with two different nanosilicate platelets (NS -F, NS+ F) at various concentrations 0 µg.mL^−1^ (**a**,**e**), 10 µg.mL^−1^ (**b**,**f**), 50 µg.mL^−1^ (**c**,**g**) and 100 µg.mL^−1^ (**d**,**h**) for 48 h. FDI (green) staining is showing live cells and PI (red) staining is showing dead cells. Scale bar is 100 µm.

Figure 5(a–h) shows the live/dead assay of hDFSCs incubated in presence of different concentrations of two nanosilicate platelets NS − F and NS + F. For live/dead assay we incubated cells for 48 h with nanosilicate concentration of 0 µg.mL^−1^ to 100 µg.mL^−1^ which is much below than the IC_50_ value of corresponding nanosilicate nanoplatelets. From Fig. 5(a–h), it is clear that most of the cells appeared alive (live cells stained by green floursecein diacetate and dead cells by red propidium iodide) correspond to all nanosilicate concentration which is similar to the control i.e., cell incubated without nanosilicates. The absence of cell death and holding spindle shaped morphology of hDFSCs in the presence of 100 µg.mL^−1^ of nanosilicates further confirms that the 100 µg.mL^−1^ is the biocompatible (nontoxic) concentration for both nanosilicate platelets (NS − F and NS + F). This observation is in well agreement with our cytotoxicity assay data Fig. 4(a–b).

### Cell proliferation

**Figure 6:**
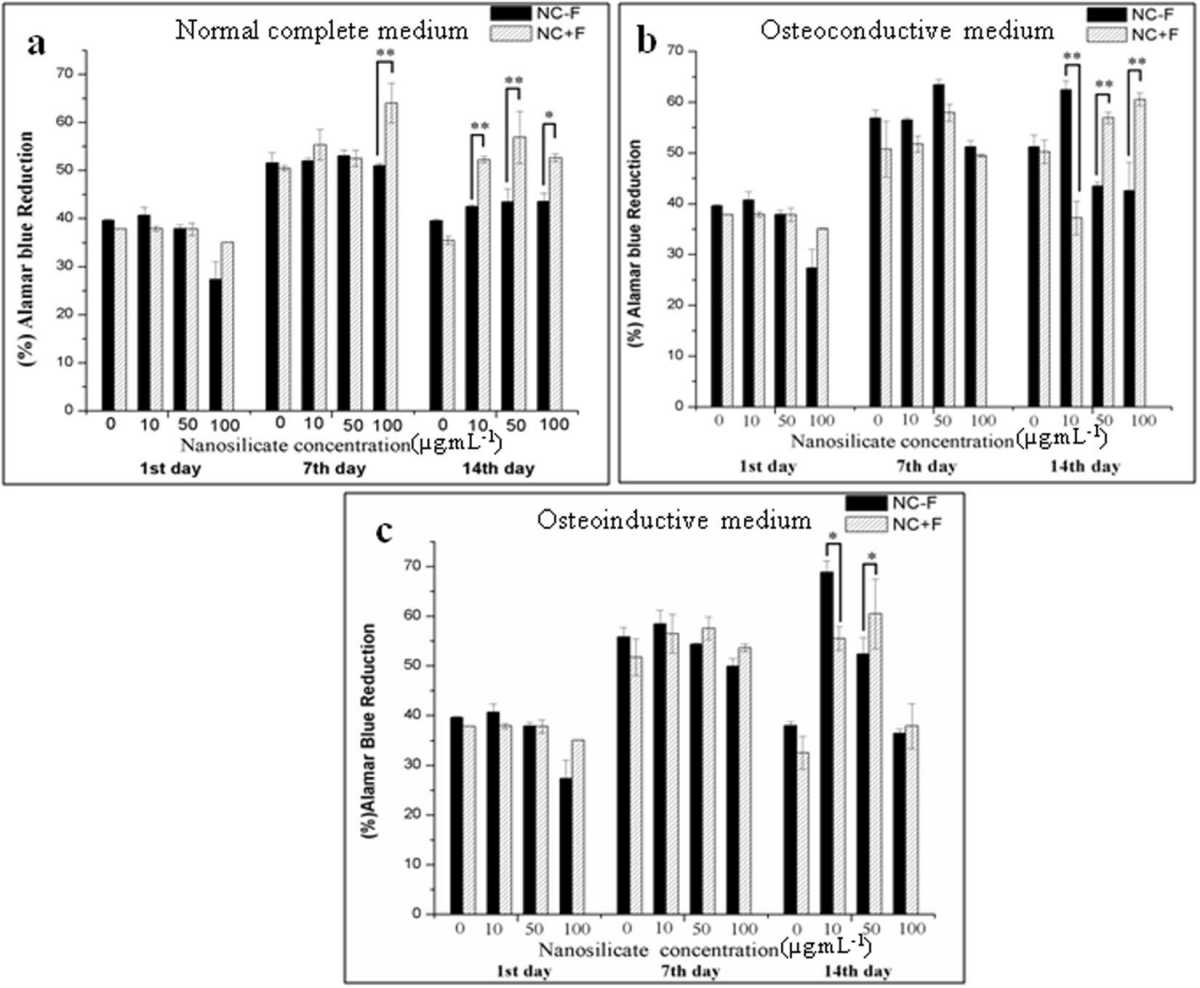
Alamar blue assay data showing proliferation of hDFSCs, incubated with variable concentration of two different nanosilicate particles (NC − F and NC + F) up to 14 d in normal complete medium (**a**), osteoconductive medium (**b**) and osteoinductive medium (**c**). (one-way ANOVA followed by Tukey post-hoc *P < 0.05, **P < 0.01, ***P < 0.001).

hDFSCs were incubated with various concentrations of NS − F and NS + F (0, 10, 50, and 100 µg.mL^−1^) over a period of 14 d to perform cellular proliferation in normal complete medium (NCM), osteoconductive medium (OCM) and osteoinductive medium (OIM). In NCM, (Fig. 6a) hDFSCs are in proliferating stage from day 1 to day 7 following slowing down in the proliferation rate at day 14 in both nanosilicate (NS − F and NS + F), possibly due to contact inhibition (cells covered the entire culture plate surface area) as well as osteogenic differentiation of cells. At day 14 cell cultured in NCM, there is a significant higher proliferation of cells in different concentrations of NS + F (10, 50 and 100 µg.mL^−1^) compared to the similar concentration of NS − F. Figure 6b shows the proliferation of hDFSCs in OCM which follows the proliferation pattern similar to hDFSCs cultured in NCM medium (Fig. 6a). NS + F (with fluoride) treated cells with concentration of 50 µg.mL^−1^ and 100 µg.mL^−1^ show higher proliferation rate at day 14 compared to non-fluoride nanosilicate, NS − F. We didn’t observe any difference in hDFSCs proliferation rate between two hDFSCs cell samples cultured in presence of 100 µg.mL^−1^ concentration of two individual nanosilicates (NS − F and NS + F) in OIM.

### Nanosilicates internalization analysis

**Figure 7:**
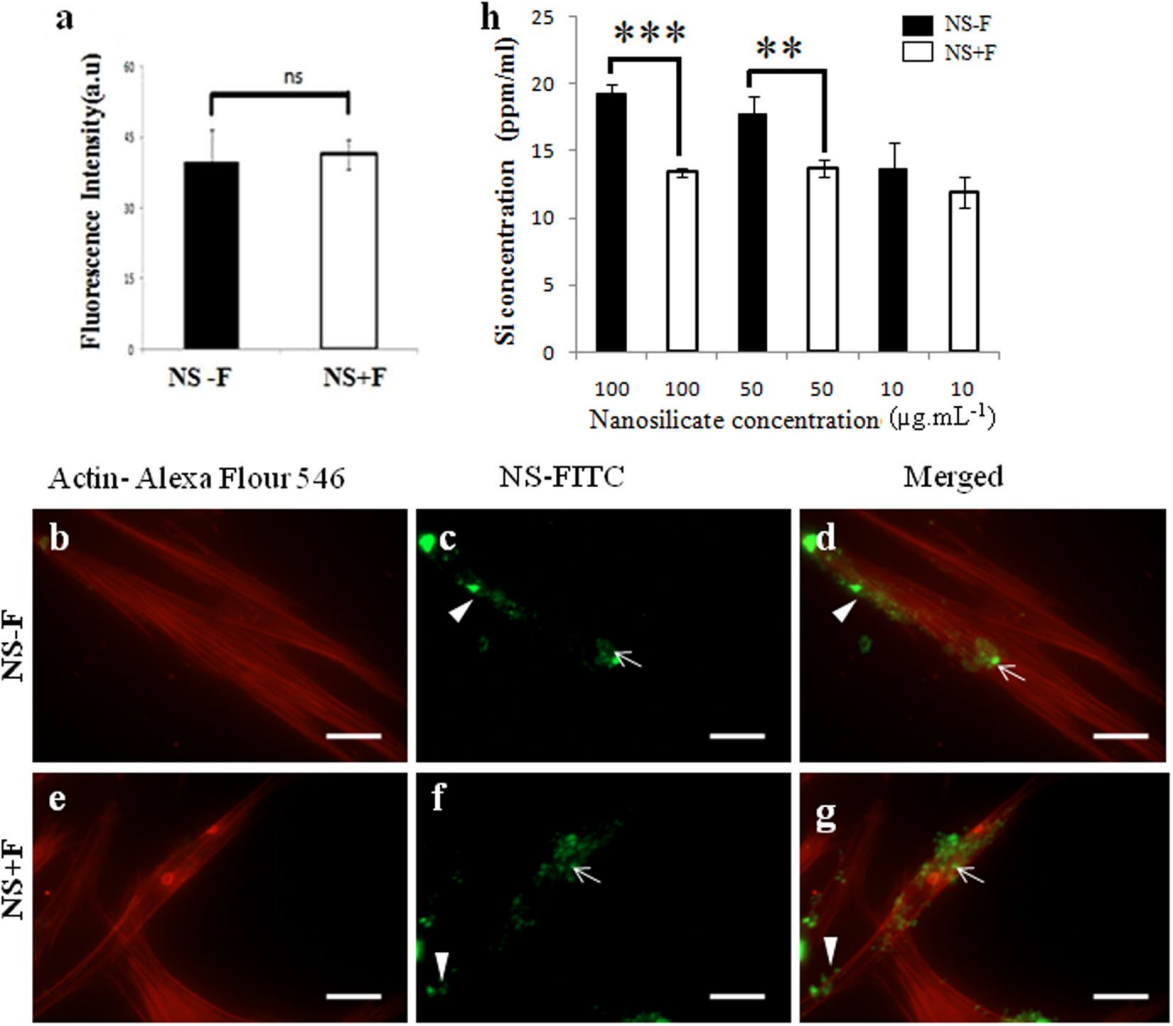
Comparative internalization of different nanosilicates (NS − F and NS + F) into the hDFSCs cultured for 48 h, measured by fluorescence microscopy (**a**–**g**) and ICP-OES method (**h**). For fluorescence imaging, hDFSCs were incubated with 50 µg.mL^−1^ FITC-NS (NS − F  and NS + F) for 48 h. Here, red channel showing cells cytoskeleton (actin filaments) stained with Alexa Flour-546 (**b**,**e**) and green channel showing nanosilicate-FITC particles (**c**,**f**), and merged channels (**d**,**g**) showing the presence of nanosilicate platelets on the cell surface (triangle) as well as inside the cells cytoplasm (arrow). Comparative internalization of two different nanosilicate particles (NS − F and NS + F) into cells measured from the fluorescence intensity of average 25 cells images (**a**). Internalization of two different nanosilicate platelets into cells when incubated in presence of various concentrations of nanosilicate particles for 48 h, measured by ICP-OES method (**h**). (one-way ANOVA followed by Tukey post-hoc *P < 0.05, **P < 0.01, ***P < 0.001, ns-statistically non-significant). Scale bar is 20 μm.

In all experiment cells were treated with respective concentration of nanosilicate platelets for 48 h followed by culture in different conditioned medium without nanosilicate particles. Nanosilicate platelets are conjugated with fluorescence (FITC) molecules to visualize and quantify the internalization of two different NS platelets using imaging technique. Figure 7 shows the nanosilicate internalization into the hDFSCs when incubated with FITC–labeled NS − F (50 µg.mL^−1^) and NS + F (50 µg.mL^−1^) for up to 48 h. Fluorescence microscopy images of cells incubated with NS platelets show attachment of nanosilicates on the cell membrane as well as presence of particles inside cell cytoplasm and perinuclear location (Fig. 7d,g) confirming the internalization of both NS − F and NS + F particles into the hDFSCs significantly. We have further quantified the amount of nanosilicates internalization into the cells by quantification of fluorescence intensity shown in the Fig. 7a. We didn’t observe significant difference in the fluorescence intensity between cell treated with NS − F and NS + F, and their internalization after 48 h of incubation. Moreover, the vesicular localization of the NS inside the cellular cytoplasm infers the active cellular transport of the nanosilicates into the cells. To further confirm this result we used ICP-OES spectroscopy to quantify internalization of NCs into hDFSCs.

Internalization of particles (silica ions concentration) measured by ICP-OES is shown in Fig. 7h, where cells were incubated in presence of various concentrations of two different nanosilicate platelets. Both nanosilicates are internalized into hDFSCs significantly but comparatively smaller size nanosilicate, NS − F (45 nm) shows relatively higher internalization compared to relatively bigger size nanoplatelet, NS + F (59 nm). In case of NS − F particles, extent of internalization increases with increase in the incubation concentration of nanosilicate, thus 100 µg.mL^−1^ concentration of NS − F results in highest internalization of nanosilicate particles into the hDFSCs. However, there is no significant increase in the internalization of NS + F when it’s concentration is increased from 10 µg.mL^−1^ to 100 µg.mL^−1^.

### Alkaline phosphatase production

**Figure 8:**
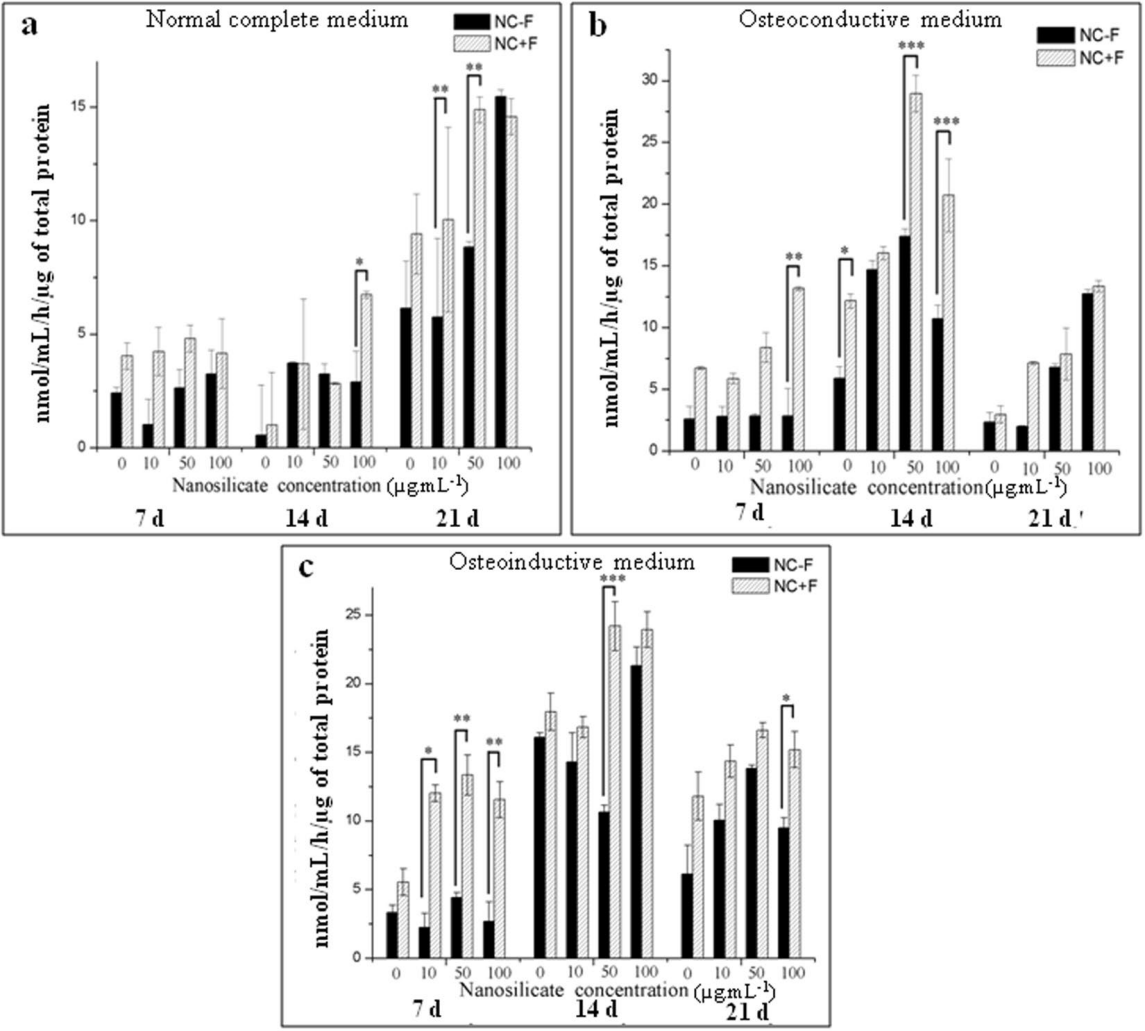
Comparative ALP activity of hDFSCs when incubated in presence of various concentration of two individual nanosilicate platelets (NS − F and NS + F) up to 21 d in three different culture medium such as NCM (**a**), OCM (**b**) and OIM (**c**). (one-way ANOVA followed by Tukey post-hoc *P < 0.05, **P < 0.01, ***P < 0.001).

Elevation of alkaline phosphatase (ALP) activity is one of the key events during osteogenic differentiation^19^, and thus hallmark for osteogenesis^20^. Figure 8 shows the ALP expression of cells when cultured with different concentrations of nanosilicates up to 21 d in three different medium (NCM, OCM and OIM). ALP expressions of cells depend on the incubation concentration of nanosilicate, incubation time and culture medium condition. For any specific nanosilicate concentration, the ALP expression of cells cultured in NCM is relatively lower (maximum 15 nmol.mL^−1^ at 21 d) than the cell cultured in OCM (maximum 30 nmol.mL^−1^ at 14 d) and OIM (maximum 25 nmol.mL^−1^ at 14 d). In the normal complete medium (NCM), cells incubated in presence of nanosilicate (NS − F or NS + F) of 50 µg. mL^−1^ and 100 µg. mL^−1^ concentration show maximum expression of the ALP at day 21 (15 nmol.mL^−1^). However, the ALP activity of cells cultured in NCM (Fig. 8a), presence of 50 nmol.mL^−1^ fluoride-nanosilicate (NS + F) is almost double (15 nmol.mL^−1^) than its non-fluoride composition, NS − F (8 nmol.mL^−1^). However there is no significant difference in the ALP expression at higher NS concentration i.e., 100 µg.mL^−1^ for both nanosilicate particles (NS − F and NS + F). The ALP expression of cells cultured in OCM and OIM, Fig. 8(b,c) reaches to peak by day 14 followed by decline in ALP activity at day 21. This trend of ALP expression is a classic feature during osteogenesis^20,21^. The hDFSCs incubated with NS + F result in higher ALP activity compared with hDFSCs incubated with non-fluoride nanosilicate (NS − F) irrespective to their days cultured, concentration of nanosilicate and medium condition. However, most significant difference in ALP expression among two different nanosilicate (with and without fluoride) incubated cell samples, is observed at 50 µg.mL^−1^ concentration of nanosilicate [Fig. 8(b,c)].

### Osteopontin expression by iimmunofluorescence staining

**Figure 9:**
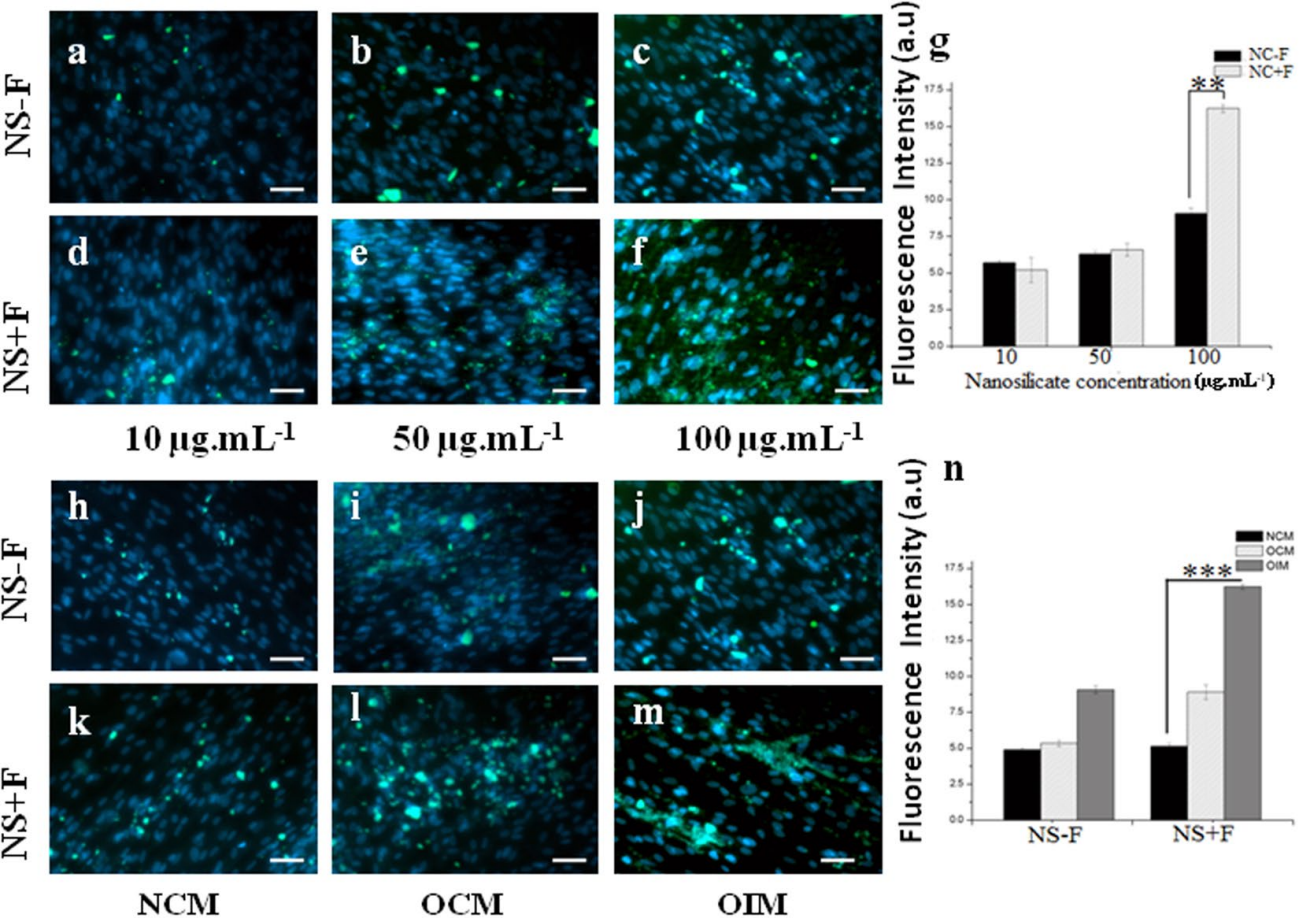
Osteopontin over expression in the hDFSCs in presence of various individual concentrations of two nanosilicates (NS − F and NS + F) for 48 h and cultured in three different medium for 14 d. hDFSC showing nucleus blue (stained by DAPI) and osteopontin green. Effect of nanosilicate concentration (10 µg.mL^−1^ to 100 µg.mL^−1^) on the osteopontin expression in hDFSCs when cultured in osteoinductive medium for 14 d (**a**–**f**) and their relative amount measured from fluorescence intensity of osteopontin expression in cells (**g**). Effect of cultured medium condition (NCM, OCM and OIM) on the osteopontin expression in hDFSCs treated with 100 µg.mL^−1^ NS concentration (**h**–**m**) and relative amount of osteopontin expression in the cells measured from fluorescence intensity (9 n). One-way ANOVA followed by Tukey post-hoc *P < 0.05, **P < 0.01, ***P < 0.001). Scale bar is 100 μm.

Osteopontin is a bone specific structural protein synthesized by maturing osteoblasts and osteocytes during bone remodeling stages^22^. The over expression of this protein is hallmark event for osteogenic differentiation of stem cells and would aid in formation of mineralization matrix formation. Figure 9 shows the immunofluorescence staining of osteopontin expression in hDFSCs, incubated with various concentrations of NS (NS − F or NS + F) particles 48 h and cultured for 14 d in three different medium, NCM, OCM, and OIM. The quantitative expression of osteopontin measured from the fluorescence images (fluorescence intensity) is depicted in the Fig. 9(g,n). Similar to ALP, osteopontin expression in the cells incubated with NS depends on the types of nanosilicate and their concentration, duration of culture and the nature of culture medium. The osteopontin expression increases with incubation concentration of NS. However, hDFSCs incubated with nanosilicate and cultured in OCM and OIM shows higher amount of osteopontin expression than the normal complete medium (NCM), Fig. 9(h,k). Moreover, cells incubated with 100 µg.mL^−1^ concentrations of nanosilicate (NS − F or NS + F) particles and cultured in OIM shows maximum osteopontin level compared to their lower concentrations (10 and 50 µg.mL^−1^) (Fig. 9g). Likewise, cells incubated with NS + F and cultured in OCM, OIM shows significanty higher expression of osteopontin compared to its non-fluoride composition, NS − F cultured in similar medium (Fig. 9n). However, among all the groups, cells incubated with 100 µg.mL^−1^ of NS + F concentration and cultured in OIM shows highest osteopontin expression. compared to NS − F.

### Mineralization studies by alizarin red staining

**Figure 10:**
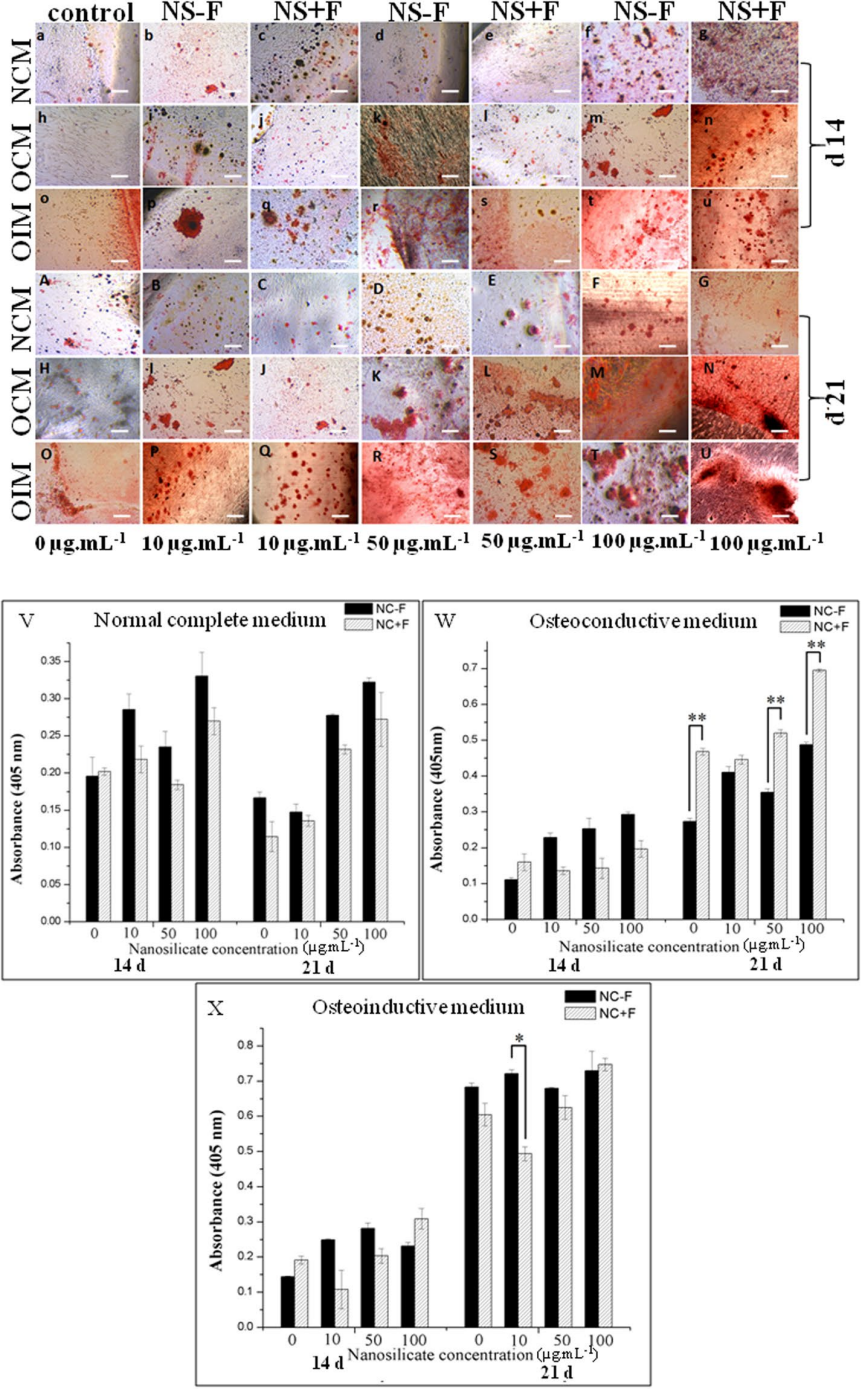
The mineralized matrix of hDFSCs stained by alizarin red. Mineralized matrix formed by hDFSCs when incubated with various individual concentration (0, 10, 50 and 100 µg.mL^−1^) of two nanosilicate (NS − F and NS + F) followed by culture in three different culture medium namely, NCM, OCM, and OIM for 14 d (**a–u**) and 21 d (**A–U**). Histograms (**V**–**X**) show the amount of alizarin red dye associated with mineralized matrix, formed by hDFSCs treated with nanosilicate particles (NS − F and NS + F) and cultured in three different medium NCM (**V**), OCM (**W**), and OIM (**X**). (one-way ANOVA followed by Tukey post-hoc *P < 0.05, **P < 0.01, ***P < 0.001).

The mineralization state, formation of inorganic calcium is studied using alizarin red staining wherein the inorganic calcium deposition acts as a marker of mineralization and osteogenesis^23,24^. hDFSCs cultured in NCM in the presence of higher concentration of both silicate platelets (NS − F or NS + F) such as 50 µg.mL^−1^ and 100 µg.mL^−1^, show mineral deposition (calcium staining by alizarin red). The quantitative data of alizarin red staining (Fig. 10V) shows relatively less amount of mineral deposition when cell are cultured in NCM in presence of both nanosilicate (NS − F or NS + F). In control cells (0 µg.mL^−1^ of NS) cultured in NCM shows very minimal nodule formation (Fig. 10a). The mineral deposition in the NS treated cells cultured in NCM indicating differentiation  of preosteoblast to osteoblast-like cells, although at lower extent than the cells cultured in OCM and OIM conditions after treatment with NS. The red alizarin staining with nodule formation evidently is seen in NS treated cells in OCM and OIM at day 14, and the staining progressively increased by day 21. In OCM, for any specific concentration of NS + F treated hDFSCs show significant higher mineralized nodules compared to its non-fluoride composition (NS − F), whereas in OIM no significant difference is observed between of NS − F and NS + F.

## Discussion

Laponite nanosilicates (NS), NS − F (Laponite XLG) and NS + F (Laponite XL21) having same crystal structure of two tetrahedral silica sheets sandwich with one Mg^2+^ containing octahedral sheet as confirmed by XRD analysis (Fig. 1). In this structure, Mg^2+^ is randomly replaced by Li^+^ leading to a net negative charge on the face of the laponite disk which is balanced by Na^+^ ions^8^. As confirmed by FTIR spectra (Fig. 2), fluoride ions (F^−^) randomly substitute ‘OH’ group in the Mg^2+^ octahedral sheet in the fluoride doped nanosilicate platelets (NS + F). In the process of dispersion in aqueous media, Na^+^ ions dissociate resulting in permanent negative charge to the faces of laponite NS as measured by negative zeta potential of NS. The presence of Mg-OH group in the octahedral sheets leads to formation of pH dependent charge on edge of the laponite disk (possess a positive charge at pH 11)^8,16,25^. At low concentration (below 1 mg.mL^−1^) NS platelets disperse as monodispersed particles in water as well as cell culture medium as visualized in the TEM and cryo-SEM Fig. 3(b,e). However, when NS particles concentration was 1 mg.mL^−1^ or more, agglomeration of the nanoplatelets into bigger clumps was visualized. Owing to their electrostatic stabilization of the colloidal nanosilicate platelets, the particle size and their colloidal stability depends on the concentration of the particles and the ionic strength of the solution^8^. Agglomeration of particles at higher concentration of NS (1 mg.mL^−1^) [Fig. 3(c,f)], attributes the formation of self-assembling “House of Cards” structure due to electrostatic interaction of negative charges on the surface of a crystal with positive charges along the edges of the other crystal^8,18^.

Recent degradation studies shown that laponite NS degrades in acidic condition^26^. Moreover, laponite are more prone to degrade in low salt medium and their low concentration dispersion, releasing degradation products such as silica [Si(OH)_4_], Na^+^, Mg^2+^, Li^+^ and F^−^ (for NS + F)^27,28^. The IC_50_ concentrations of NS − F particles for hDFSCs are close to the reported IC_50_ value of NS − F with other cell such as hBMSC^18^. However, the IC_50_ value of fluoride-nanosilicates NS + F at 24 h of incubation is double than NS − F, showing more biocompatibility nature of fluoride-nanosilicate platelets (NS + F) over their non-fluoride-nanosilicate (NS − F). At increase in the concentration beyond 1000 µg.mL^−1^ the electrostatically stabilized nanosilicate platelets agglomerate into larger particles [in Fig. 3(c,f)]. The possible covering of the entire cell membrane by this large agglomerated particles, thus hampering the cellular or metabolic activities of cells, may result in cytotoxicity of NS at higher concentration. At present few hydroxyapatite formulations, approved by FDA have been using in clinic for bone tissue regeneration^29^ [It is important to compare the toxicity of NS with hydroxyapatite particles. The IC_50_ values of nanosilicate platelets are higher than the IC_50_ values of hydroxyapatite nanoparticles (250 µg.mL^−1^)^30^, and silica nanoparticles (500 µg.mL^−1^)^31,32^ which infers about the superior cytocompatibility nature of nanosilicate platelets. In Fig. 4(d,h), hDFSCs retained their long and spindle fibroblastic morphology (similar to the control without nanosilicate) below the NS (both NS − F and NS + F) concentration 1000 µg.mL^−1^, further confirming the biocompatibility concentration of NS. Relative decrease in the hDFSCs densities above 1000 µg.mL^−1^ NS (Fig. 4e,i), and rounded morphology at 5000 µg.mL^−1^ NS, infer the inhibition of cellular functionality and toxicity of nanosilicates (Fig. 4f,j). The concentrations used for the live/dead assay (0–100 µg.mL^−1^) are well below the toxic concentration. Hence there are very few dead cells and most of the cells retained spindle shaped morphology confirming 100 µg.mL^−1^ is the biocompatible (nontoxic) concentration for both nanosilicate platelets (NS − F and NS + F). Thus, we used concentration up to 100 µg.mL^−1^ of both nanosilicates for all other study (internalization and differentiation).

Internalization of nanosilicates is important to probe whether the nanosilicate transport into the hDFSCs and interact with cell/cellular components at nanoscale which may effect on their proliferation and osteogenic differentiation. It has already been reported that nanoclay enters into the cells through clathrin-mediated pathway^9,10^ and the internalization efficiency depends on the size of the particles, surface charge and concentration of nanoparticles^9^. Owing to the difference in size between NS − F (45 nm) and NS + F (59 nm), we could expect difference in extent of internalization of these nanosilicate into hDFSCs. The degree of internalization of two different nanosilicates determined from the fluorescence intensity of internalized nanosilicates does not show any significant difference (Fig. 7a). To confirm this result we have further performed ICP-OES where silica ion concentration is measured. The Fig. 7h shows relatively higher internalization of NS − F into hDFSCs compared to NS + F. The relative smaller size of NS − F (45 nm) may be attributed for their higher internalization^33^. As expected, the degree of internalization of nanosilicate into hDFSCs increases with increasing their concentration (10 µg.mL^−1^ to 100 µg.mL^−1^). However, the effect of nanosilicate concentration in their internalization into hDFSCs is very prominent for NS − F than NS + F. The high internalization of nanosilicate into the hDFSCs confirms the possible interaction of nanosilicate with cells in the nanoscale which may affect their cellular function such as proliferation and differentiation.

The increase in proliferation of hDFSCs in the presence of NS + F may be attributed to the presence of fluoride ions and their conductive factors in their proliferation. However, in OIM and higher nanosilicate concentration (50 µg.mL^−1^ and 100 µg.mL^−1^), cell proliferates until day 7 of culture following declines in the proliferation at day 14 (Fig. 6c). The relative decrease in proliferation of hDFSCs at day 14 of culture may be attributed to their osteogenic differentiation, resulting from the cumulative effect of NS and osteogenic inducing factors from medium.

hDFSCs incubated with NS + F result in higher ALP activity compared with hDFSCs incubated with non-fluoride nanosilicate particles (NS − F) irrespective to their days of cultured and concentration of nanosilicate. However, most significant difference in ALP expression of hDFSCs among two different nanosilicate (with and without fluoride), is observed at nanosilicate concentration of 50 µg.mL^−1^, Fig. 8(b,c). This may be due to additive effect arising from osteogenic inducing factors within the culture medium, and activation of innate osteoinductive factors from the leached ions from nanosilicates (NS − F and NS + F). It is important to note that the cells are only incubated with nanosilicates for 48 h and then the cells are thoroughly washed with PBS. The internalization studies show the significant internalization of nanosilicate (NS − F; NS + F) within 48 h of incubation. We noticed that even though the NS + F internalization are comparatively lower than NS − F, it aids in osteogenic induction higher than NS − F. The over expression of ALP in the cells treated with nanosilicate cultured in normal growth medium confirm the nanosilicate mediated osteogenic differentiation of hDFSCs which is further confirmed by osteopontin and mineralized matrix deposition. This also signifies that these nanosilicates can support and aid in osteogenesis in the absence of inducing growth factors in the medium. However, the significant higher ALP expression for fluoride-nanosilicate (NS + F) incubated cells possibly explained by fluoride ion mediated enhancement of osteogenic differentiation and osteoblast proliferation^34^, as the affects of fluoride ions are more pronounced on osteoblast precursors rather than on the mature osteoblasts^35^. It has been reported that ALP levels increased with fluoride ion exposure (1.0 ppm) for 1 week in human osteoblast-like MG-63 cells^36^. Hence this signifies the bioactive nature of these nanosilicate by inducing osteogenic effects on hDFSCs and the fluoride-dopped NS + F shows higher osteogenic inducing properties than the nonfluoride nanosilicate (NS − F).

The nanosilicate mediated over expression of osteopontin may be attributed that NS aid in providing physicochemical cues necessary for osteogenic induction of progenitor cells. Higher concentration (50 µg.mL^−1^ to 100 µg.mL^−1^) of NS would be more favourable for osteoinduction. Moreover, these NS may provide sites for mineralization. It is reported that fluoride down-regulates TGF-β1 signaling^37^, and also up regulates bone mineralization by enhancing osteoblast differentiation^24^, and stimulating alkaline phosphatase (ALP) activity and OPN, a matrix protein found in mineralized tissues and plays a pivotal role in modulating osteoclastic activities. These *in vitro* studies have demonstrated that NS have an anabolic effect on enhancement of hDFSCs differentiation to osteoblasts. However, the osteoinductive effect seems to be more prominent in fluoride-nanosilicate (NS + F) compared to non-fluoride nanosilicate (NS − F).

Stem cells differentiated into osteoblasts is known to deposit mineralizing nodules in culture^38^. Among the two NS, NS + F shows higher osteoinductive effect and we could expect higher extent of mineralization for cell treated with NS + F compared to NS − F. Additionally, the higher mineralization in NC + F compared to NC − F may be attributed to the presence of fluoride ions in low concentrations in the mineralization solution promoting the hydrolysis of calcium phosphate to apatite transformation. Moreover, fluoride ion complexes with calcium and sodium and may present as a composite of calcium sodium fluoride species^39^. This fluoride nanosilicates (NC + F) provides essential stimulating factors for positive effect on hDFSCs *in vitro*, promoting osteoblast differentiation and mineralization for bone tissue formation.

## Conclusion

Nanosilicate platelets with fluoride doping (NS + F) are used for systematic evaluation of their interaction with human dental follicle stem cells (hDFSCs) to probe the cytotoxicity, cellular transport (internalization) and osteogenic differentiation capabilities in comparison with already reported nanosilicates platelets without fluoride (NS − F). The *in vitro* toxicity of these nanosilicate shows the biocompatibility nature of both nanosilicate particles. The *in vitro* differentiation assays proved that these nanosilicates posses inherent osteogenic inducing properties. These inducing properties are enhanced when they are used in the presence of OCM and OIM. Both nanosilicate (NS − F and NS + F) shows significant osteogenic properties to the hDFSCs. However, NS + F treated cells show higher ALP activity, osteopontin protein levels and significant alizarin red staining compared to NS − F treated cells. NS + F consisting of fluorosilicate would have paved way for higher osteoinducing properties. The fluoride-doped nanosilicate platelet is a next generation nanobiomaterials having high osteogenic potential for tissue engineering application such as periodontal regeneration. BMP-2 molecules have been shown as most potent osteoinductive molecules for stem cell (including hBMSCs) osteogenic differentiation and bone formation^40^. The promising results in this studies further infer that the fluoride nanosilicate platelets could be used as a substitute of expensive growth factors such as BMP-2, FDA approved most potent osteoinductive molecules currently in clinical use for alveolar bone regeneration (INFUSE Bone Graft, Medtronic, USA). However, it is important to validate higher osteogenic potential of NS + F compared to NS − F *in vivo* which is currently under progress. This is the first study to present the information demonstrating the effect of compositional variation of nanosilicates on cellular bioactivity and their osteogenic induction potential.

## Experiment Section (Materials and Methods)

### Materials

Synthetic Laponite nanosilicates (NS), Laponite XLG (NS − F) and Laponite XL21 (NS + F), were kindly gifted by BYK, USA. Minimal essential medium –alpha modification (alpha MEM), fetal bovine serum (FBS), penicillin/streptomycin/antibiotic solution, Trypsin-EDTA, Phalloidin, fluorescein diacetate (FDA) and propidium iodide (PI), and alamar blue were purchased from Invitrogen, USA. BSA, β-glycerophosphate, ascorbic acid, dexamethasone, formaldehyde, Triton-X 100, FITC albumin, alizarin red S, Oil-O Red, and para-nitrophenyl phosphate substrate with glycine buffer were purchased from Sigma-Aldrich, USA. BCA Protein Assay Kit and Glacial acetic acid were purchased from Thermo Fisher Scientific, USA, and SRL, India. Anti-osteopontin, donkey antigoat IgG conjugated with Alexa Flour 488 purchased from Abcam, USA. DAPI, Alcian Blue was purchased from Alpha Aesar, USA. MTS assay kit was purchased from Promega, USA. Milli-Q water (18.2 MΩ.cm) was used wherever needed. All chemicals were used with no further modifications and purification.

### Dental follicle stem cell (hDFSCs) isolation and culture

Human noncarious third molar dental follicles were collected from tooth buds of healthy patients (Male, Age 27), that underwent extractions for orthodontics reasons at Apex Multispecialty Center, Hyderabad, after obtaining informed consent from patient. All experiments were conducted in accordance with the requirements of the Institutional Ethics Committee (IEC) and Institutional Committee for Stem Cell Research (IC SCR) of Indian Institute of Technology, Hyderabad. All experimental protocol related to hDFSCs (extraction, isolation, culture, and experiment with nanosilicate) in this study were approved by IEC and IC SCR, Indian Institute of Technology, Hyderabad. Follicle tissue was digested for 1 hour at 37 °C in agitation in a solution of 3 mg.mL^−1^ collagenase-type 1, obtained single cell suspensions were seeded in a 25 cm^2^ culture flask and cultured with α-MEM supplemented with 10% fetal bovine serum (FBS), 100 U.mL^−1^ penicillin-G, 100 μg.mL^−1^ streptomycin and 0.25 μg.mL^−1^ fungizone. Flask was incubated at 37 °C and 5% CO_2_ and the medium was changed every 3 d (see Supporting Information for more details).

Phenotypic expression studies were performed to characterize the hDFSCs for mesenchymal stem cell (MSCs) specific markers. For phenotypic analysis, cells (hDFSCs at Passage 3) were trypsinized and sequentially stained with fluorescent tagged antibodies using MSC phenotypic kit (MACS-Miltenyi Biotech). hDFSCs cultivated in 10% FBS supplement showed high positivity for CD 73, CD 90, CD 105, and were found negative (CD-14, CD-20, CD-34 and CD-45) (see Supporting Information for more details).

To study the multipotential capacity of hDFSCs (passage 3) were differentiated into osteogenic, dipogenic and chondrogenic lineages using suitable differentiating culture medium for 3 w (see Supporting Information for more detials). Differentiation of hDFSCs into osteogenic, adipogenic and chondrogenic lineages were confirmed by formation of specific tissue formation such as bone, fat and cartilage respectively (see Supporting Information for more details).

### Characterization of NS and their preparation of suspension

X-ray diffraction pattern (XRD) of two NS samples was performed to determine their crystal structure. For XRD pattern of dry NS samples was recorded by Bruker D8. Advance diffractometer using Cu Kα radiation (λ = 1.54 Å). Diffraction intensities were measured from 20° to 80° of 2θ angels at a scan speed of 1.2° min^−1^ and increments of 0.02°. To determine the bonding nature in two NS, the FT-IR spectra was recorded usingFTIR, TENSOR 37, Bruker. The NS sample (10 mg) was mixed with 1 gm of dry KBr powder and pressed into a thin pellet by applying 5000–10000 psi pressure. The pallet was used to record FT-IR spectra in the range of 400 to 4000 cm^−1^ with 2 cm^−1^ resolution.

Stock solution of silicate nanoplatelets were prepared by dissolving the suitable amount of nanosilicate into distilled water with vortexing for 10 mins. Later silicate suspensions were subjected to ultrasonication (Sonics, Vibra-Cell™ Ultrasonic Liquid Processors, VCX 130, USA 25% Amplitude for 2 min) to allow complete dissolution of platelets into the solution. These solution were analysed for determination of the morphology and size of the nanosilicates by transmission electron microscopy (TEM-JEM 2100) and Cyo-SEM (JEOL,JSM-7600F). The nanosilicate platelets stock solution of 1 mg.mL^−1^ concentration were prepared by dissolving in alpha MEM medium and subjected to ultrasonication (25% Amplitude for 2 min). This stock solution of nanosilicate suspensions were used to make different concentration of nanosilicate in the complete medium for further *in vitro* experiments.

### hDFSCs treatment with nanosilicates platelets

The hDFSCs were seeded into 48 wells plate with 10,000 cells/well and cultured for 24 h. After 24 h, the hDFSCs were treated with different concentration (0 µg.mL^−1^, 10 µg.mL^−1^, 50 µg.mL^−1^, 100 µg.mL^−1^) of nanosilicates (NC − F and NC + F) for 48 h. After 48 h of incubation, the silicate containing media was replaced with the corresponding normal complete medium (NCM), osteoconductive medium (OCM) and osteoinductive (OIM) media. Osteoconductive media was supplemented with β-glycerophosphate 20 mmol.L^−1^ and ascorbic acid 50 µmol.L^−1^ whereas osteoinductive media was supplemented with β-glycerophosphate 20 mmol.L^−1^, ascorbic acid 50 µmol.L^−1^ and dexamethasone 100 nmol.L^−1^. Cells were incubated with normal and osteoconductive, and osteoinductive media without nanosilicates used as negative controls in these studies.

### Cytotoxicity assay

Cytotoxicity of nanosilicates was determined using MTS assay. Pre-seeded hDFSCs (10000 cells/well) in 96 well plates were incubated with different concentration (0 µg.mL^−1^ to 5000 µg.mL^−1^) of NS − F and NC + F in NCM for 72 h. MTS assay was performed as described by the manufacturer’s protocol (Promega assay Kit, USA). IC_50_ (half maximal inhibitory concentration) concentration at which hDFSCs metabolic activity decreased to 50% were determined using this assay. In short, culture media was removed and replenished with 200 µL of fresh culture medium, and 10 µL of MTS solution added to each well. Samples were incubated at 37 °C for 4 h. Absorbance at 409 nm was measured using a microplate reader (Perkin Elmer, USA). Later the IC_50_ values of both of these nanosilicate platelets were determined by fitting the dose response curve.

### Cellular morphology

The effect of different concentrations of NS − F and NC + F on the cell morphology and cytoskeleton organization were studied by incubating the pre-seeded hDFSCs (5000 cells/well in 48-well plate) with different concentration of NS. After 48 h, the media was removed and the cells were washed with PBS twice and fixed with 3.7% formaldehyde in PBS, followed by three times PBS washing. Subsequently the cells were treated with 0.1% Triton-X 100 in PBS. The actin filaments (F-actin) were stained with Phalloidin Alexa Fluor 546 (2 µL.mL^−1^ of 1% BSA in PBS) for 60 min, and the nuclei with DAPI (3 µL.mL^−1^ of 1% BSA in PBS). After the incubation, cells were washed thrice with PBS. The plates were stored in dark, at 4 °C until imaged. Imaging was performed by using a fluorescence microscop (Carl Zeiss, Germany using Axiovert-Zen soft ware).

### Live/dead assay

hDFSCs (10^4^ cells) were seeded onto each well of 48 well plates, followed by 48 h incubation in normal complete medium (500 µL) with different concentration of (NC − F and NC + F) (0 µg.mL^−1^ to 100 µg.mL^−1^) at 37 °C in a 5% CO_2_ incubator. After incubation, the medium was removed and cells were washed with 1X PBS thrice and fixed with 3.7% formaldehyde in PBS. Live and dead hDFSCs were qualitatively distinguished by sequential staining with fluorescein diacetate (FDA) and propidium iodide (PI). They were incubated in staining solution (5 mg.mL^−1^ of FDA and 2 mg.mL^−1^ of PI in α-MEM) for 5 min and washed with 1X PBS twice. The cells were visualized under a fluorescence microscope.

### Metabolic activity assay (Alamar Blue)

The metabolic activity of hDFSCs was determined using Alamar Blue Assay. hDFSCs (10^4^ cells) were seeded onto each well of 48 well plate, followed by 48 h incubation (37 °C and 5% CO_2_) in normal complete medium containing different amount of NS (NC − F and NC + F) (0 µg.mL^−1^ to 100 µg.mL^−1^). After incubation, the medium was removed and cells were washed with 1X PBS. These nanosilicates incubated hDFSCs were further cultured in three different media such as NCM, OCM, and OIM for 14 d. The assay was performed on the day 1, 7, and 14, according to the manufacturers protocol. In brief, after predetermined duration, the media was removed and the cells were washed twice with PBS. Then the PBS was replaced with 10% (v/v) of alamar blue reagent in complete medium and incubated at 37 C, 5% CO_2_ for 3 h. After incubation, the supernatant of the cultures was aliquoted into 96-well plate in triplicates and fluorescence reading was recorded using a microplate reader (Perkin Elmer, USA) (570 nm excitation and 600 nm emission wavelengths).

### Nanosilicate internalization into hDFSCs by imaging method

Fluorescence tagged (NC − F and NC + F) were prepared by mixing FITC-albumin (1 mg.mL^−1^) with nanosilicate particles and incubated for 24 h. The unbounded FITC-albumin was removed by dialysis. Pre-seeded hDFSCs were treated with 50 µg.mL^−1^ of nanosilicates (NC − F and NC + F) suspension in the normal complete medium and incubated for 48 h at 37 °C and 5% CO_2_. After incubation, the medium was removed and cells were washed with 1X PBS thrice, and fixed with 3.7% formaldehyde in PBS, followed by washing twice using PBS. Subsequently the cells were treated with 0.1% Triton-X 100 in PBS. The actin filaments (F-actin) were stained with Alexa Fluor 546 Phalloidin (2 µL.mL^−1^ of 1% BSA in PBS) for 60 min. Later cellular uptake of NS into hDFSCs was visualized and quantified by fluorescence microscopy. Amount of internalization was calculated using ZEN PRO Intensity image analysis software.

### Inductively coupled plasma optical emission spectroscopy (ICP-OES)

The concentrations of nanosilicate particles internalized into hDFSCs were also measured by ICP-OES (Bruker Aurora M90). In this measurement after incubation of cells with nanosilicate particles (as described above), cell supernatant was removed and washed three times with PBS. Cells were trypsinized and carefully transferred into 15 mL centrifuged tube. Cells were lysed using 1% Triton-X 100 for 30 min at room temperature followed by digestion using 500 μL of concentrated nitric acid for 24 h. The digested cell samples were diluted by 3 mL of double distilled water, and subjected to ICP-OES analysis. The concentration of Si in the digested solution was measured. At least 3 wells in 24 well plate were incubated with only medium during internalization studies and performed similar cell digestion process (as described above). This digestion samples (without cells) were used as control to rule out any silica contribution from the media/reagent/water used for cell culture and digestion steps. All experiments were performed in triplicate. For all measurements, the ICP-OES instrument was initialized, optimized and standardized using the manufacturer’s recommendations.

### Alkaline phosphatase production

Alkaline phosphatase (ALP) activity of hDFSCs incubated with different amount of NS (0 µg.mL^−1^ to 100 µg.mL^−1^ of NC − F and NC + F) and cultured media (NCM, OCM, and OIM) was measured to determine the osteogenic potential. ALP activity was measured using a colorimetric endpoint assay which quantified the conversion of p-nitrophenol phosphate (pNPP) to yellow p-nitrophenol (pNP) by ALP enzyme. After culture of hDFSCs for specific days (7, 14, and 21), cells were lysed with Triton-X 100 (0.5% Triton-X I00 in PBS) and freeze/thawed three times to disrupt the cell membranes. 50 μL of the cells lysate and 150 μL of the liquid substrate (pNPP) with glycine buffer was added in each well of 96 wells plate and incubated in the dark for 60 min. The absorbance was measured at 405 nm after adding 20 μL of stop solution (2 M NaOH) in a microplate reader. A standard curve was made using standards ALP (0 to 20 μmol). Amount of ALP in the samples were determined from the standard curve. ALP activity was normalized to the total protein content of the cells. Total protein was determined using BCA Protein Assay Kit according to manufacturer’s protocol.

### Osteopontin expression by immunofluorescence staining

The expression of osteogenic marker protein such as osteopontin (OPN) was measured by immunofluorescence. Osteopontin expressions of hDFSCs treated with various concentrations of NS (NS − F and NS + F) and cultured in different media (NCM, OCM, and OIM) for 14 d, were analyzed by immunostaining method. Briefly, after specific days in culture, the cells were fixed with 4% paraformaldehyde and permeabilized with 0.5% Triton-X 100, and blocked with 3% bovine serum albumin. Then the samples were incubated with the osteopontin (Abcam, USA) primary antibodies as per manufacturers recommended dilution (1: 200) for 30 min at 37 °C in dark followed by incubation with secondary antibodies (donkey antigoat IgG, Abcam, USA) conjugated with Alexa Flour 488 for 30 min. All the samples were counterstained for nuclei using DAPI and analyzed under fluorescence microscope.

### Alizarin Red S staining

The hDFSCs treated with different amount of NC − F and NC + F (0 µg.mL^−1^ to 100 µg.mL^−1^) were cultured in different media such as NCM, OCM, and OIM. After specific days (14 and 21) in culture, extend of mineralization was determined using alizarin red assay. After cultureding for  d 14 and 21, cells were fixed with 3.7% formaldehyde for 20 min, and then washed three times with PBS followed by addition of 2% (w/v) alizarin red S solution. After 10 min incubation at room temperature, the excess of alizarin red was washed with distilled water for several times. The alizarin red stained on the samples was imaged using optical microscopy [Olympus (CKX-53) inverted microscope]. The amount of alizarin stained was estimated by colorimetric assay. In this assay, 10% acetic acid was added to the each well. After 2 h of incubation, the dye solution were transferred to tubes and centrifuged to remove the cell debris. The supernatant was removed and neutralized with 10% ammonium hydroxide. 200 μL of each dye sample was added to 96-well plates and the absorbance (405 nm) was recorded using a microplate reader.

### Statistical analysis

All experiments were done in triplicate and expressed as mean ± standard deviation. For all experiments, the statistical significance between different groups was determined by one-way ANOVA with the Tukey post hoc test using “Instat GraphPad” software for statistical somputation, The groups with significant difference was represented with symbols (*P < 0.05, **P < 0.01, ***P < 0.001).

## Supplementary information

Supplementary Information
