# Supplementary material for: Effect of Fluoride Doping in Laponite Nanoplatelets on Osteogenic Differentiation of Human Dental Follicle Stem Cells (hDFSCs)
Source: Sci Rep. 2019 Jan 29;9:915. doi: 10.1038/s41598-018-37327-7 (PMC6351553; doi:10.1038/s41598-018-37327-7)
Supplement: Supplementary file 1 — Supplementary Information [file 41598_2018_37327_MOESM1_ESM.docx]

**“Effect Of Fluoride Doping In Laponite Nanoplatelets On Osteogenic Differentiation Of Human Dental Follicle Stem Cells (hDFSCs)”**

Induvahi Veernala ^a #^, Jyotsnendu Giri ^a #^*, Arpan Pradhan ^b^, Poulomi Polley ^a^, Ruby Singh ^a^, Sunil Kumar Yadava^a^

a. Department of Biomedical engineering, Indian Institute of Technology Hyderabad , Kandi , Telangana, India

b. Department of Biosciences and Bioengineering, Indian Institute of Technology Bombay, Powai, Mumbai, Maharashtra, India

# Both authors equally contributed

**hDFSCs isolation and Expansion of hDFSCs**

Teeth were obtained from patients (healthy donors) undergoing extraction. Immediately after extraction, the third molars were washed using 0.2% chlorhexidine and phosphate buffered saline (PBS) followed by a wash with sterile distilled water. Dental follicle tissue surrounding the tooth is retrieved with a scalpel blade under sterile conditions inside laminar hood . The dental follicle tissue was thoroughly disaggregated with scissors and enzymatically digested with Collagenase type-1 at 37^o^C for 60 minutes. After incubation, the enzymatic digestion was stopped by adding normal complete media (composed of alpha-MEM with 2mM L-glutamine), supplemented with 10% FBS and 100 μM L-ascorbic acid 2-phosphate 100 units/ml penicillin,100 μg/ml streptomycin and the cell suspension were mixed thoroughly and centrifuged at 800 g for 10 minutes. The pellet obtained was resuspended in complete medium and then incubated at 37°C in 5% CO2. The medium change was given thrice a week to propagate the DFSCs to confluency. The cells were passaged at 80-85% confluency by trpsinising (Trypsin-EDTA 0.5%.). Part of cell suspension were utilized for experiments and remaining cells were frozen with 5:4:1 freezing mix [FBS+ Alpha MEM+ DMSO] and stored at -80 ^0^C for future use.


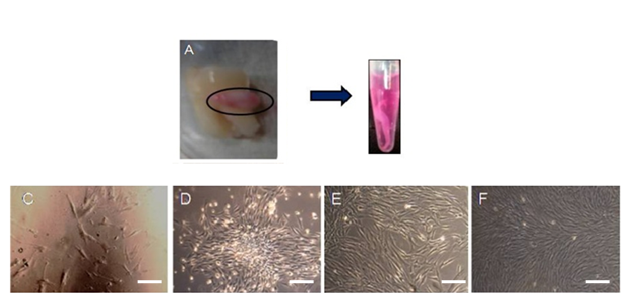


B

Figure SI 1. Showing optical microscopy images of primary culture of hDFSCs. Third (III) molar tooth (A). Dental follicle after digestion (B). Scattered cells seen with fibroblastoid features after 72 hours of hDFSC (C) cultures. hDFSC colonies (D) seen after 7 days of culture. Cellular morphology stabilization achieved after 10 days in hDFSC (E),. Culture with total confluence seen of DFSC( F) cultures. Scale bar is 100 µm.

**Characterization**

To study the multipotential capacity of dental stem cells, Passage 3 cells of hDFSCs were differentiated into osteogenic , adipogenic and chondrogenic lineages for 3 weeks of *invitro* culture. A total of 1x10^4^ cells of hDFSCs were seeded into each well of a 48 well plate.

**Osteogenic differentiation**

For the induction of osteoblastic differentiation, cells were grown in an osteogenic medium consisting of α-MEM supplemented with 10% FBS, 10 nM dexamethasone and 50 µg/ml ascorbic acid , 20 mM β –glycerophosphate for 21 days . Flasks were incubated at 37° C and 5% CO2 and the medium was changed every 3 days. After 21 days, cells were washed twice in PBS after being fixed in 3.7 % formaldehyde for 10 min and then incubated in 0.1% alizarin red solution in Tris Hcl (pH 8.3) at 37^o^C for 5 min. After being washed twice in PBS, cells were routinely observed and photographed under a light microscope (Olympus CKX 53).

**Adipogenic Differentiation**

hDFSCs were cultured in adipogenic medium consisted of alpha MEM supplemented with, 0.5μM methylisobutylxanthine , 100nM Dexamethasone and 50μM indomethacin . Medium was changed every 3 days. After 21 days, the cells growing under adipogenic conditions were washed twice with PBS followed by fixation in 3.7 % formaldehyde for 10 mins and then incubated with 0.2% Oil red O stain for 5 mins, later plates were rinsed well with distilled water, cells were routinely observed and photographed under a optical microscope .

**Chondrogenic differentiation**

hDFSCs are cultured in alpha MEM supplemented with 10^−7^ M dexamethasone , 1 μM ascorbate-2-phosphate , and 10 ng/ml transforming growth factor-beta 1 (TGF-β1). After 21 days, the cells growing under chondrogenic conditions were washed twice with PBS ,fixed in 3.7 % formaldehyde for 10 mins and incubated in alcian blue (Alcian Blue is a colorimetric dye that can detect the presence of sulfated glycosaminoglycans deposition of cells, indicative of a chondrogenic lineage) staining for 5 mins, later plates were rinsed well with distilled water, cells were routinely observed and photographed under a optical microscope.

**Phenotypic Expression Studies**

Phenotypic expression studies were performed to characterize the hDFSCs for mesenchymal stem cell (MSCs) specific markers. For phenotypic analysis, cells (DFSCs at Passage 3) were trypsinised and sequentially stained with fluorescent tagged antibodies using MSC phenotypic kit (MACS-miltenyi) and were analysed by flourescence activated cell sorer -FACS( BD FACS ARIA III)). hDFSCs cultivated in 10 % FBS supplement showed high positivity for CD 73 , CD 90, CD 105 and were found negative for CD-14/20/34/45.


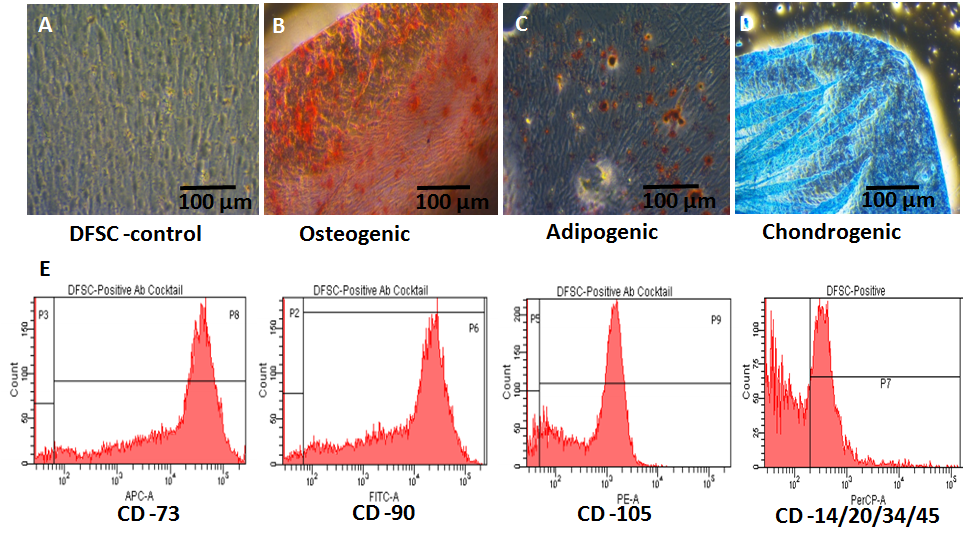


Figure SI 2. Showing multipotential nature of hDFSCs. Optical microscope image of hDFSCs (A) culture without any differentiation media and considered as the negative control. Optical microscopy images of hDFSCs differentiation towards osteogenic (B) and adipogenic differentiation (C), Chondrogenic differentiation (D) lineages after 21 days of culture. Cell differentiation in osteogenic induction medium confirmed by presence of calcium deposits stained with alizarin red (B), adipogenic differentiation, confirmed by presence of lipid droplets stained with oil red O (C), like wise chondrogenic differentiation confirmed by staining of GAGs with Alcian blue. Positive and negative phenotypic markers expression (E) of hDFSCs evaluated by FACS analysis revealed that hDFSCs show high positivity for CD 73 , CD 90, CD 105 and were found negative for CD14/CD20/34/45.
